# A three-dimensional hybrid electrode with electroactive microbes for efficient electrogenesis and chemical synthesis

**DOI:** 10.1073/pnas.1913463117

**Published:** 2020-02-12

**Authors:** Xin Fang, Shafeer Kalathil, Giorgio Divitini, Qian Wang, Erwin Reisner

**Affiliations:** ^a^Department of Chemistry, University of Cambridge, CB2 1EW Cambridge, United Kingdom;; ^b^Department of Materials Science & Metallurgy, University of Cambridge, CB3 0FS Cambridge, United Kingdom

**Keywords:** *Geobacter*, electrogenesis, electrosynthesis

## Abstract

Integration of electroactive bacteria into electrodes combines strengths of intracellular biochemistry with electrochemistry for energy conversion and chemical synthesis. However, such biohybrid systems are often plagued with suboptimal electrodes, which limits the incorporation and productivity of the bacterial colony. Here, we show that an inverse opal-indium tin oxide electrode hosts a large population of current-producing *Geobacter* and attains a current density of 3 mA cm^−2^ stemming from bacterial respiration. Differential gene expression analysis revealed *Geobacter*’s transcriptional regulations to express more electron-relaying proteins when interfaced with electrodes. The electrode also allows coculturing with *Shewanella* for syntrophic electrogenesis, which grants the system additional flexibility in converting electron donors. The biohybrid electrode containing *Geobacter* can also catalyze the reduction of soluble fumarate and heterogenous graphene oxide, with electrons from an external power source or an irradiated photoanode. This biohybrid electrode represents a platform to employ live cells for sustainable power generation and biosynthesis.

## Significance

Addressing the global challenge of sustainability calls for cost-effective and eco-friendly pathways to go beyond the existing energy-intense synthetic routes. Biohybrid electrochemical systems integrate electroactive bacteria with synthetic electrodes to leverage the power of biocatalysis for energy conversion and chemical synthesis. This work presents a three-dimensional electrode scaffold to couple the intracellular metabolism with extracellular redox transformations by means of electrochemistry. The large population of bacteria actively metabolizing within the electrode scaffold produces a benchmark current density. The biohybrid electrode can also carry out synthetic reactions within or beyond biochemical pathways driven by solar light. This hierarchical electrode provides a robust and versatile platform to wire bacteria’s intrinsic physiological functionalities with artificial electronics for sustainable energy conversion and chemical production.

Interfacing the biocatalytic machinery of live cells with synthetic electrodes provides a cross-disciplinary approach for sustainable energy production and chemical synthesis (1, 2). While an array of biocatalysts are already being employed in synthetic chemistry (3), microorganisms have demonstrated unrivalled synthetic potential due to sequences of well-tuned biosynthetic routes and the advancing techniques of synthetic biology, which allows selective synthesis of complex chemicals from the simplest feedstocks (e.g., CO_2_, H_2_O) under physiological conditions (4, 5). Of particular interest are electroactive bacteria such as *Geobacter* and *Shewanella* that have evolved unique mechanisms to discharge respiratory electrons by reducing insoluble Fe(III) or Mn(IV) oxides (6). These bacteria can transport endogenous electrons across insulating and impermeable cell envelopes to extracellular electron acceptors via outer membrane c-type cytochromes (OMCs), conductive bacterial nanowires, and/or self-secreted flavins (7). Their ability to exchange electrons with inorganics via transmembrane electron conduits couples intracellular metabolism with extracellular redox transformations (8, 9), and allows a biohybrid system to exploit the biological metabolism via artificial electronics for electrogenesis and chemical synthesis (10).

The biohybrid systems rely on electrodes that can host a colony of electroactive bacteria with intact metabolic pathways (11). Electrodes also allow probing and controlling the bacteria’s physiological functionalities with electrochemical methodologies. Carbon-based electrodes, such as graphite and carbon cloth, are broadly applied in microbial fuel cells owing to their electrochemical stability, biocompatibility, and structural plasticity (12). Nevertheless, the architecture of these electrodes is commonly not optimized for a large population of bacteria while ensuring effective diffusion of nutrients and dissipation of wastes (11). In addition, their hydrophobic surfaces are not conducive to electrical interaction with hydrophilic bacteria (13). Therefore, sessile bacteria on such electrodes tend to form compact biofilms with sluggish electron transfer and inefficient mass transport, which engender adverse stresses limiting their proliferation and productivity (14).

The hallmark of *Geobacter sulfurreducens* is its current-producing capability in microbial fuel cells (6). Its ability to metabolize organic pollutants and precipitate soluble heavy metals renders it also potentially applicable in bioremediation (15). Moreover, its complete genome sequence primes transcriptome analysis to probe its regulation strategies to maintain cellular homeostasis under various conditions (16).

**Fig. 1.**
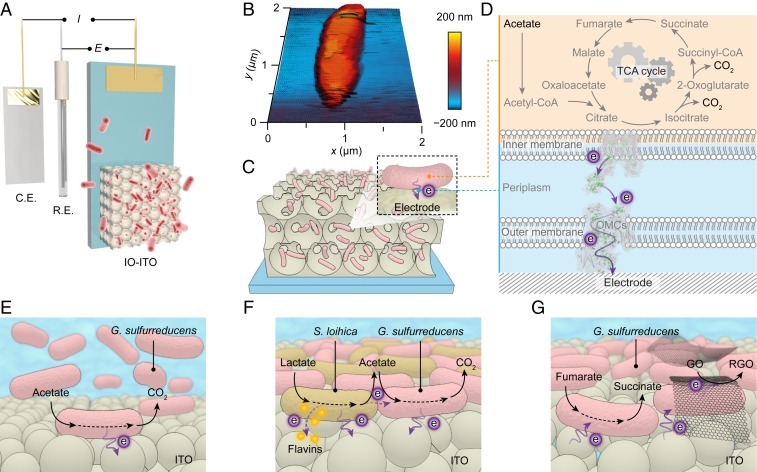
Schematic representation of microbial electrogenesis and electrosynthesis within the IO-ITO electrodes. (*A*) An IO-ITO|*G. sulfurreducens* electrode is assembled into a three-electrode system with a counter electrode (C.E.) and a reference electrode (R.E.). (*B*) Atomic force microscopy (AFM) image of *G. sulfurreducens* on a silicon wafer. (*C*) Schematic representation of a biohybrid electrode where *G. sulfurreducens* colonized on the IO-ITO scaffold. (*D*) Extracellular electron transfer at the interface between *G. sulfurreducens* and an electrode. Acetate is metabolized into CO_2_ via the TCA cycle and excess electrons are discharged to an external electrode via OMCs. (*E*) Schematic representation of microbial electrogenesis. *G. sulfurreducens* is respiring on an electrode surface with acetate as the electron donor while continuously releasing electrons to the electrode. (*F*) Syntrophic electrogenesis where *S. loihica* metabolizes lactate into acetate and transfers electrons to the electrode mainly through self-excreted flavins. *G. sulfurreducens* then consumes acetate and releases electrons to the electrode. (*G*) Microbial electrosynthesis of succinate and RGO using a biohybrid IO-ITO electrode. At negative potentials, the sessile *G. sulfurreducens* exploits exogenously supplied electrons to maintain its metabolism while transferring excess reducing equivalent to soluble fumarate and heterogeneous GO.

Here we employ an inverse opal-indium tin oxide (IO-ITO) electrode as a platform for microbial electrogenesis and electrosynthesis using *G. sulfurreducens* (Fig. 1 *A* and *B*). ITO is hydrophilic and the porous electrode architecture provides easy access for bacteria penetration and colonization (Fig. 1*C*). When positive potentials are applied, planktonic *G. sulfurreducens* from the medium solution attaches on the electrode surface. The sessile bacteria metabolize acetate to support its growth through the tricarboxylic acid (TCA) cycle, while discharging excess electrons to the electrode via OMCs, which is registered as a continuous anodic current (Fig. 1 *D* and *E*). Transcriptome analysis by RNA sequencing revealed that *G. sulfurreducens* regulates gene expression in order to respire on electrodes. Furthermore, *Shewanella loihica* PV-4 was introduced together with *G. sulfurreducens* on the IO-ITO electrode to achieve syntrophic electrogenesis by linking their metabolic pathways (Fig. 1*F*), which will grant the system additional flexibility in using different electron donors. Electrosynthesis was carried out by poising negative potentials on the resulting IO-ITO|*G. sulfurreducens* electrode. Under such conditions, *G. sulfurreducens* accepts electrons from the electrode to sustain its metabolism and disposes respiratory electrons by reducing soluble fumarate or heterogeneous graphene oxide (GO) (Fig. 1*G*). Lastly, to outsource the electron supply to a renewable source, the biohybrid electrode was coupled with a photoanode to achieve photoelectrosynthesis without applying an external electrochemical bias.

## Results and Discussion

### Microbial Electrogenesis.

**Fig. 2.**
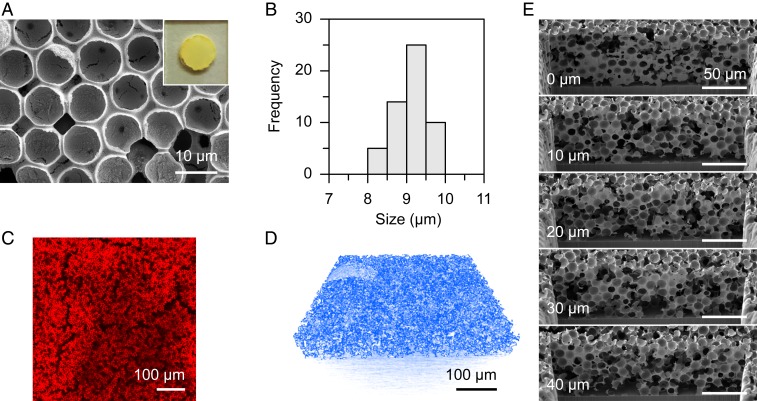
Structure of the IO-ITO electrode. (*A*) Top-view SEM image. *Inset* shows a photograph of the electrode (*S* = 0.25 cm^2^). (*B*) Histogram of the pore size distribution of the IO-ITO electrode. (*C*) CLSM image of the IO-ITO electrode, showing channels that allow bacteria to penetrate. A total of 20 μL of rhodamine B solution (5 mM, in methanol) was dropcast on an IO-ITO electrode and dried in the dark. Excitation: 552 nm. Emission: 590 to 640 nm. (*D*) X-ray microscopy image of the interconnected IO-ITO scaffold (colored in blue, see Movie S1). (*E*) Serial cross-sectional SEM images of the IO-ITO electrode acquired from FIB-SEM. Cross-sectional views of every 10 μm are displayed (Movie S2).

IO-ITO electrodes were prepared by a coassembly method using 10-μm polystyrene beads as the structural template and ITO nanoparticles (average size: 50 nm) as the electrode material to suit the dimension of *G. sulfurreducens* (length: 1.5 to 2 μm; diameter: 400 to 500 nm) (*SI Appendix*, Fig. S1 and Fig. 1*B*) (17, 18). The resulting electrode features interconnected macropores (8 to 10 μm) accessible to bacteria and a mesoporous skeleton permeable to both nutrients and products (Fig. 2 and Movies S1 and S2). The IO-ITO electrode had a geometrical area of 0.25 cm^2^ and a film thickness of ∼60 µm (Fig. 2 *A* and *E*).

**Fig. 3.**
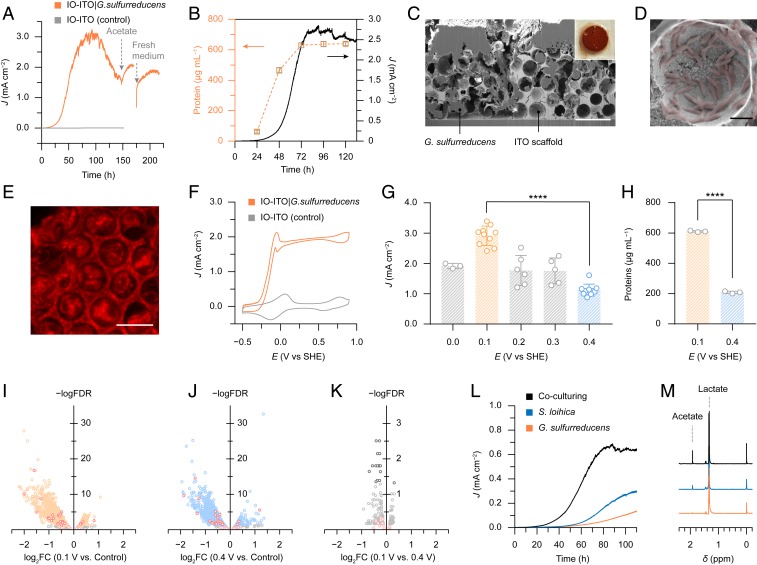
IO-ITO electrodes as the platform to accommodate electroactive bacteria for microbial electrogenesis. (*A*) A representative current of *G. sulfurreducens* respiring inside an IO-ITO electrode at 0.1 V vs. SHE with acetate (40 mM, pH 7.4). A bare IO-ITO electrode was used as a control. The two arrows indicate the addition of 40 mM acetate to the existing medium and the replenishing of a fresh medium containing 40 mM acetate, respectively. (*B*) Colorimetric quantification of proteins in the hybrid electrodes during bacterial colonization at 0.1 V vs. SHE (a typical current output shown as the black trace). (*C*) Cross-sectional SEM image of an IO-ITO|*G. sulfurreducens* electrode. (Scale bar: 20 μm.) The *Inset* shows a photograph of the electrode (*S* = 0.25 cm^2^). (*D*) *G. sulfurreducens* (artificially colored in red) attached on the surface of an IO-ITO electrode. (Scale bar: 2 μm.) (*E*) CLSM image of an IO-ITO|*G. sulfurreducens* electrode. The hybrid electrodes were stained with 5-cyano-2,3-ditolyl tetrazolium chloride (CTC, 10 mM) and incubated in the dark for 30 min at 25 °C. Excitation: λ_ex_ = 488 nm, emission: λ_em_ = 600 to 650 nm. (*F*) Representative CV scans of an IO-ITO|*G. sulfurreducens* electrode and a bare IO-ITO electrode (control) with acetate. The redox wave near 0 V vs. SHE is derived from the medium solution. Scan rate: 5 mV s^−1^. (*G*) Potential dependence of the current produced by IO-ITO|*G. sulfurreducens*. Independent samples: 0.0 V: *n* = 3; 0.1 V: *n* = 10; 0.2 V: *n* = 6; 0.3 V: *n* = 6; 0.4 V: *n* = 10. (*H*) Colorimetric quantification of proteins in IO-ITO|*G. sulfurreducens* electrodes prepared at 0.1 V and 0.4 V vs. SHE. *n* = 3 independent samples. Error bars represent the standard error of the mean. Significance value: ****P < 0.0001. (*I*–*K*) Volcano plots of differential gene expression of *G. sulfurreducens* in IO-ITO electrodes at 0.1 V and 0.4 V vs. SHE. (*I*) 0.1 V vs. control; (*J*) 0.4 V vs. control; (*K*) 0.1 V vs. 0.4 V. The control group was the planktonic *G. sulfurreducens* anaerobically cultured in a medium solution with 20 mM acetate and 50 mM fumarate (pH 7.2) at 30 °C. The expression difference is represented by the log fold change in base 2 (log_2_FC) versus a baseline group (*I* and *J*, control; *K*, 0.4 V). The expression difference is considered significant provided that the false discovery rate (FDR), the adjusted *P* value for multiple testing, is less than 0.05 (−logFDR > 1.3). Positive logFC values represent higher expression compared with the baseline group. The red points indicate the genes encoding putative c-type cytochromes in *G. sulfurreducens* identified by ref. 16. Each point represents the average value of one transcript in three replicates. (*L*) Representative currents of *G. sulfurreducens*, *S. loihica*, and a mixed culture of *G. sulfurreducens* and *S. loihica*, with an IO-ITO electrode at 0.4 V vs. SHE with lactate (40 mM, pH 7.4). (*M*) ^1^H NMR spectra of the electrolyte solution extracted after 100 h electrogenesis with IO-ITO|*G. sulfurreducens*, IO-ITO|*S. loihica*, and IO-ITO|mixed cultures. TMSP-d^4^ (1 mM) was used as the reference (0 ppm) and internal standard for quantification. ^1^H NMR peaks of acetate (singlet, 1.92 ppm) and lactate (doublet, 1.34 ppm) are indicated. All of the electrochemical experiments were performed under a N_2_:CO_2_ atmosphere (80:20, v:v%) at 30 °C.

*G. sulfurreducens* was integrated on an IO-ITO scaffold from the electrolyte solution by applying a potential of 0.1 V vs. standard hydrogen electrode (SHE). During this process, planktonic *G. sulfurreducens* penetrated into the electrode scaffold and metabolized acetate into CO_2_ while discharging electrons to the electrode (Fig. 1 *C* and *D*). Bacteria then proliferated and progressively colonized the entire electrode, producing an increasing anodic current that plateaued at 3 mA cm^−2^ after 80 h (Fig. 3*A*), which corresponds to a volumetric current density of 500 mA cm^−3^. This volumetric current density represents a benchmark performance in microbial electrogenesis and approaches the volumetric current limit (1,000 mA cm^−3^) of a single bacterium (*SI Appendix*, Table S1) (19). Control experiments show that the recorded current was exclusively derived from bacterial metabolism (*SI Appendix*, Figs. S2 and S3), making it a good proxy for the bacteria’s metabolic activity. Quantification of the proteins inside the hybrid electrode supported that the growth of bacteria aligned with the increase of current density (Fig. 3*B*). The high current density is attributed to the IO-ITO electrode architecture, which compartmentalized bacteria colonies with a conductive and permeable scaffold, and thus allowed a large population of bacteria to actively metabolize therein. In contrast, *G. sulfurreducens* on a flat ITO-coated glass and a flat gold electrode produced substantially less current (∼0.2 mA cm^−2^) and therefore yielded a much thinner biofilm (<5 µm) (*SI Appendix*, Fig. S4) (14). The current started decaying in the wake of acetate depletion, which can be partially restored by supplementing acetate into the current medium or replenishing with a fresh medium containing acetate (Fig. 3*A*).

The resulting IO-ITO|*G. sulfurreducens* electrode displayed a typical reddish color stemming from the redox-active multihaem c-type cytochrome (Cyt *c*) (Fig. 3*C*, *Inset*). Focused ion beam-scanning electron microscopy (FIB-SEM) imaging shows that the bacteria penetrated through the entire IO-ITO electrode and were in close contact with the mesoporous scaffold (Fig. 3 *C* and *D*, *SI Appendix*, Fig. S5, and Movie S3). Confocal laser scanning microscopy (CLSM) images manifest the respiratory activity of living bacteria and indicate that bacterial viability was well-retained in the electrode scaffold (Fig. 3*E* and *SI Appendix*, Fig. S6).

Under turnover conditions, the IO-ITO|*G. sulfurreducens* electrode exhibited a characteristic sigmoidal cyclic voltammetry (CV) trace with an onset potential of −0.25 V vs. SHE (Fig. 3*F*, *E*_CO_2_/acetate_ = −0.29 V vs. SHE, pH 7.0) (20). The CV profile points to a typical catalytic response of a biofilm, where the catalytic current is limited by the extracellular electron transport via OMCs (21). This is further evidenced by a control experiment that suppressed Cyt *c* production in bacteria by limiting the iron availability during growth, without affecting the bacterial viability (*SI Appendix*, Fig. S7 *A*–*C*) (22). The iron-depleted (ΔFe) *G. sulfurreducens* produced negligible current (0.5 μA cm^−2^) and a nonturnover CV wave (*SI Appendix*, Fig. S7 *D*–*F*), which confirms the necessity of Cyt *c* for microbial electrogenesis. Electrons delivered from the bacterium were transferred through the conducting IO-ITO scaffold. A control experiment with an insulating IO-ZrO_2_ scaffold on an ITO-coated glass produced negligible current and no bacterial colony was formed (*SI Appendix*, Fig. S8), which demonstrates that the conductivity of the electrode scaffold is essential for the bacterium’s outward electron transfer and biofilm formation.

### Potential-Dependent Electrogenesis.

The plateau anodic current varies with the applied electrochemical potential (Fig. 3*G* and *SI Appendix*, Fig. S9). The highest current density was attained at 0.1 V vs. SHE (2.9 ± 0.1 mA cm^−2^, *n* = 10), whereas it reduced to 1.1 ± 0.1 mA cm^−2^ (*n* = 10) at 0.4 V vs. SHE (Fig. 3*G*). Colorimetric protein quantification revealed that the biohybrid electrode at 0.1 V vs. SHE contained more proteins than at 0.4 V vs. SHE (Fig. 3*H*). We thus infer that bacteria can overcome the thermodynamic challenge arising from a lower electrochemical potential to discharge electrons outward by adopting a different set of pathways (23). This would allow them to maintain competitive advantages in habitats where redox states of electron acceptors are frequently varying due to environmental and meteorological perturbations. It is common practice in the field to apply high potentials (e.g., 0.4 V vs. SHE) to establish an electron sink for microbial respiration (*SI Appendix*, Table S1), but our results suggest that such positive potentials might not be optimal for microbial electrogenesis.

RNA sequencing was then employed to understand whether the culturing in electrodes and potential difference can induce transcriptional responses. *G. sulfurreducens* for RNA extraction was collected after the bacteria ceased proliferation in the electrodes at different potentials (after the current plateau) and in a planktonic solution with fumarate (in the stationary phase) (*SI Appendix*, Figs. S3*B* and S10). Differential gene expression analysis shows a substantial down-regulation of gene expression when bacteria are grown on electrodes, compared with those cultured in a medium solution with fumarate as the electron acceptor (Fig. 3 *I* and *J* and *SI Appendix*, *Supplementary Text*). This observation agrees with a previous study using graphite as the electrode and Fe(III) citrate as the soluble electron acceptor (24). It suggests that *G. sulfurreducens* deployed a different metabolic strategy that consumes less energy when interfaced with an electrode (24, 25). The transcriptional regulation is likely to occur during the initial lag phase (Fig. 3*A*), during which electron transfer pathways are shifted to favor an insoluble electron acceptor (electrode) (24). Nevertheless, there was no significant change in gene expression at different potentials (0.1 V and 0.4 V vs. SHE) (Fig. 3*K*), despite large differences in current density (Fig. 3*G*). These findings imply that *G. sulfurreducens* adjusted its gene expression to keep intracellular metabolism in tune with physiological needs with different electron acceptors, whereas electrode potentials cannot induce tangible responses at a transcriptional level. The question of how *G. sulfurreducens* can sense electrode potentials and respond to potential variations remains unclear and requires further investigations (26, 27).

### Syntrophic Electrogenesis.

In nature, different bacteria form symbiotic partnerships via interspecies mass transport or electron transfer to overcome environmental disadvantages (28). This inspires a syntrophic strategy for electrogenesis, which employs the syntrophy between mixed cultures of electroactive bacteria, and thus grants the system additional resilience to environmental perturbations such as limited electron donors. *S. loihica* is an electrogenic bacterium ubiquitously thriving in aquatic and sedimentary environments. It has evolved robust sensing and regulatory systems that confer its metabolic versatility (29). *S. loihica* and *G. sulfurreducens* have similar morphology and dimensions (*SI Appendix*, Fig. S11), but differ in metabolic pathways: *S. loihica* utilizes lactate as the carbon and energy resource instead of acetate and it engages with extracellular electron acceptors mainly through self-secreted flavins (30). By coculturing *S. loihica* and *G. sulfurreducens* in an IO-ITO electrode, lactate can be used as the sole electron donor to support the electrogenesis of both strains. In this case, *S. loihica* metabolizes lactate into acetate that can be further utilized by *G. sulfurreducens*, while both bacteria release electrons to the electrode (Fig. 1*F*). Such a syntrophic pathway can increase the stoichiometric production of electrons and further attest that the IO-ITO electrode is a robust and versatile host for various microbial communities.

As *G. sulfurreducens* poorly utilizes lactate for metabolism (31), the current output at 0.4 V vs. SHE (0.13 mA cm^−2^) with lactate was far below that with acetate as the electron donor (1.07 mA cm^−2^) (Fig. 3*L*). This is also evidenced by a reduced *G. sulfurreducens* population on the electrode (*SI Appendix*, Fig. S12*A*). The current density produced by *S. loihica* (0.30 mA cm^−2^) with lactate was smaller than that of *G. sulfurreducens* with acetate at 0.4 V vs. SHE, despite a large population of *S. loihica* on the electrode (Fig. 3*L* and *SI Appendix*, Fig. S12*B*). This results from a diffusion-governed extracellular electron transfer by *S. loihica* (32), which is kinetically less efficient compared with direct electron transfer via OMCs in *G. sulfurreducens*. Inoculation of both *S. loihica* and *G. sulfurreducens* attained a higher current of 0.68 mA cm^−2^, and yielded more acetate (∼2.9 mM) than *S. loihica* alone (∼1.6 mM) (Fig. 3 *L* and *M*). These together point to a syntrophy between *G. sulfurreducens* and *S. loihica*: the presence of *G. sulfurreducens* perhaps assisted *S. loihica* in discharging more electrons via interspecies electron transfer (33), which produced more acetate and facilitated the growth of both strains (*SI Appendix*, Fig. S12*C*).

### Microbial Electrosynthesis.

**Fig. 4.**
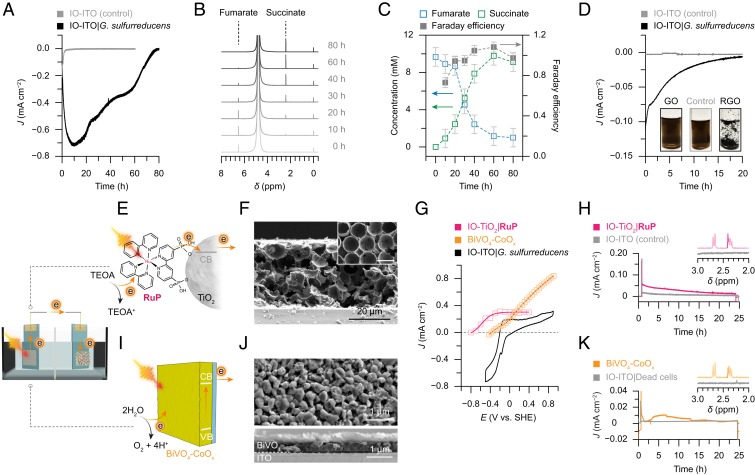
Microbial electrosynthesis and photoelectrosynthesis with IO-ITO|*G. sulfurreducens* electrodes. (*A*) Representative cathodic current of an IO-ITO|*G. sulfurreducens* electrode catalyzing fumarate (10 mM, pH 7.4) reduction at −0.45 V vs. SHE. A bare IO-ITO electrode was used as a control. (*B*) ^1^H NMR spectra of the electrolyte solution aliquoted during the course of reaction. TMSP-d^4^ (1 mM) was used as the reference (0 ppm) and internal standard for quantification. ^1^H NMR peaks of fumarate (singlet, 6.52 ppm) and succinate (singlet, 2.41 ppm) are indicated. (*C*) Quantification of reactants and products and Faraday efficiency during the course of reaction. (*D*) Cathodic current of an IO-ITO|*G. sulfurreducens* electrode reducing GO (0.1 mg mL^−1^) at −0.3 V vs. SHE. A bare IO-ITO electrode was used as a control. The *Inset* shows photographs of GO solutions before (labeled “GO”) and after reduction by a bare IO-ITO (labeled “Control”) and an IO-ITO|*G. sulfurreducens* electrode (labeled “RGO”). All of the reactions were performed in a N_2_:CO_2_ atmosphere (80:20 v:v%) at 30 °C, with Pt and Ag/AgCl as counter and reference electrode, respectively. (*E*) Schematic representation of a PEC cell consisting of an IO-TiO_2_|**RuP** anode and an IO-ITO|*G. sulfurreducens* cathode. Under irradiation, the excited RuP* dye injects an electron into the conduction band of the TiO_2_ electrode, which is further directed to the cathode via an external circuit. The **RuP**^**+**^ dye is regenerated by extracting an electron from TEOA. (*F*) SEM image of an IO-TiO_2_ electrode. The *Inset* shows the top view of the electrode (Scale bar: 10 µm.) The IO-TiO_2_ electrode has a thickness of 40 µm and macropore size of 10 µm. (*G*) Photocurrent from chronoamperometry of the IO-TiO_2_|**RuP** (0.25 cm^2^) and BiVO_4_-CoO_x_ (1.0 cm^2^) photoanodes (plotted at different applied potentials) and cyclic voltammogram of the IO-ITO|*G. sulfurreducens* electrode in fumarate (10 mM, pH 7.2) solution. Three-electrode configuration, scan rate: 5 mV s^−1^. (*H*) Light-driven fumarate reduction with an IO-TiO_2_|**RuP**||IO-ITO|*G. sulfurreducens* two-electrode system at zero bias. A bare IO-ITO electrode without bacteria was used as the cathode for a control experiment (gray trace). TEOA (25 mM, in 50 mM KCl) was used as the electron donor for the photoanode. (*I*) Schematic representation of a PEC cell consisting of a BiVO_4_-CoO_x_ anode and an IO-ITO|*G. sulfurreducens* cathode. BiVO_4_ absorbs light and donates excited electrons to the external circuit while oxidizing water with the aid of the CoO_x_ cocatalyst. (*J*) Top-view (*Top*) and cross-sectional (*Bottom*) SEM images of a BiVO_4_-CoO_x_ electrode. The thickness of BiVO_4_ film was 500 nm and CoO_x_ cocatalysts were deposited on top. (*K*) Light-driven fumarate reduction with a BiVO_4_-CoO_x_||IO-ITO|*G. sulfurreducens* two-electrode system at zero bias. A hybrid electrode with dead bacteria (deactivated by 0.1% glutaraldehyde) was used as the cathode for a control experiment (gray trace). A PBS solution (20 mM Na_2_HPO_4_, pH 7.3) was used for the photoanode compartment. The *Insets* in *H* and *K* are ^1^H NMR spectra of the solution extracted from the cathode compartment after 24 h of irradiation. TMSP-d^4^ (1 mM) was used as the reference (0 ppm) and internal standard for quantification. The NMR peak of succinate (singlet, 2.41 ppm) is highlighted and the doublet peak at 2.7 ppm is assigned to malate. Conditions: 20 mM fumarate, pH 7.2, *U* = 0 V, *I* = 100 mW cm^−2^, AM 1.5G, in a N_2_:CO_2_ (80:20 v:v%) atmosphere at 25 °C. The photocurrent was normalized to the geometrical area of the cathode (0.25 cm^2^).

Electrosynthesis was carried out by poising a negative potential on the biohybrid electrode that was cultured at 0.1 V vs. SHE for 80 to 100 h, until the current stabilized. In this case, *G. sulfurreducens* receives electrons to sustain its metabolism and disposes excess reducing equivalents to reduce chemicals (20). We employed a prototypical reaction, fumarate reduction, to exemplify the potential of leveraging intracellular metabolism for chemical synthesis. Fumarate reduction to succinate is part of a biosynthetic pathway that transforms CO_2_ into organics and is an essential reaction for bacterial survival under anaerobic conditions (34). At −0.45 V vs. SHE, the IO-ITO|*G. sulfurreducens* electrode generated a cathodic current that returned to zero after 80 h (Fig. 4*A*). During the process, fumarate was stoichiometrically reduced to succinate with a Faraday efficiency of (93 ± 12)% (Fig. 4*C*), whereas fumarate cannot be electrochemically reduced by a bare IO-ITO electrode at the same potential (Fig. 4*A*) (20).

We further explored reactions beyond the bacteria’s native metabolic pattern. Planktonic *G. sulfurreducens* can reduce GO by extracellularly transferring electrons to GO in the presence of electron donors (35). The sessile *G. sulfurreducens* in an IO-ITO scaffold reduces GO in a similar fashion at −0.3 V vs. SHE (Figs. 1*G* and 4*D*). The reduction of GO after 20 h is indicated by the increasing hydrophobicity of reduced GO (RGO) and rise of intensity ratio of D and G bands in the Raman spectra (Fig. 4*D* and *SI Appendix*, Fig. S13) (36). In the absence of bacteria, a minimum cathodic current was recorded (Fig. 4*D*), suggesting GO was reduced by *G. sulfurreducens* and not by the IO-ITO scaffold at −0.3 V vs. SHE (conventional electrochemical GO reduction is implemented at a more negative potential; −0.7 V vs. SHE at pH 7.2) (37). We therefore show the synthetic versatility of the biohybrid electrode to prepare functional materials beyond natural metabolites with reduced energy input under physiological conditions to rival more energy-intense synthetic routes.

### Microbial Photoelectrosynthesis.

We coupled an IO-ITO|*G. sulfurreducens* electrode with a photoanode to outsource the electron supply to photochemistry. We employed an IO-TiO_2_ photoanode (geometrical surface area: 0.25 cm^2^) sensitized with a photosensitive phosphonated [Ru^II^(2,2'-bipyridine)_3_]-based dye (denoted as **RuP**, λ_max_ = 457 nm) to enable visible-light absorption (Fig. 4 *E* and *F*) (38, 39). The onset potential of the IO-TiO_2_|**RuP** photoanode in the presence of triethanolamine (TEOA, pH 7.2) was determined at −0.6 V vs. SHE (Fig. 4*G* and *SI Appendix*, Fig. S14*A*), whereas the catalytic wave of fumarate reduction by the biohybrid electrode appeared at −0.2 V vs. SHE (Fig. 4*G*). The energy levels were thus well aligned to allow autonomous light-driven fumarate reduction without an electrochemical bias in two-electrode configuration (*SI Appendix*, Fig. S14*B*). After 24 h of simulated solar irradiation (*I* = 100 mW cm^−2^, Air Mass 1.5 Global [AM 1.5G]), 0.79 ± 0.10 mM succinate was detected, along with intermediate metabolites such as malate (doublet, 2.7 ppm), pyruvate (singlet, 2.38 ppm) (8), corresponding to a succinate yield of (7.8 ± 1.1)% (Fig. 4*H*). The presence of additional metabolites indicates that the bacteria retained their metabolic activity with electrons supplied by the photoanode and thus reduced fumarate via intracellular biosynthetic sequences.

The IO-TiO_2_|**RuP** photoanode employs a sacrificial reagent (TEOA) as the electron donor and is prone to photodegradation (38). To overcome these drawbacks, we resorted to monoclinic BiVO_4_ as the light-absorbing material in light of its well-suited band structure for water oxidation to O_2_ (band gap: 2.4 eV; conduction band potential: −0.4 V vs. SHE, pH 7.0) (40). We employed BiVO_4_ deposited with a CoO_x_ cocatalyst as the photoanode to directly extract electrons from water (in a phosphate buffer solution, pH 7.3) (Fig. 4 *I* and *J*) (41). The BiVO_4_-CoO_x_ electrode displayed a photocurrent onset potential at −0.35 V vs. SHE (Fig. 4*G* and *SI Appendix*, Fig. S14*C*) and the BiVO_4_-CoO_x_ photoanode therefore generated a smaller current at zero bias in a two-electrode configuration with 0.51 ± 0.20 mM succinate being detected after 24 h of irradiation (*I* = 100 mW cm^−2^, AM 1.5G) (Fig. 4*K* and *SI Appendix*, Fig. S14*D*). Inactivation of the bacteria by biocide on the cathode resulted in no succinate and other metabolites, confirming fumarate reduction was performed through bacterial metabolism (Fig. 4*K*).

Light-driven fumarate reduction has been previously carried out using isolated flavoenzymes as the biocatalyst, but the system performance was highly limited by the fragility of isolated enzymes and susceptible to enzyme orientations that dictate the electron transfer at biointerfaces (42, 43). The microbial system here enabled higher catalytic capacity and improved stability, thanks to the large number of robust bacteria integrated inside the IO-ITO scaffold. Moreover, the proteinaceous electron conduits on bacterial membranes allow for omnidirectional electron transfer toward electrodes, regardless of the orientation of the bacteria. This photosynthetic system decouples light harvesting on the photoanode from chemical transformation at the cathode, rendering the system optimization flexible.

## Conclusion

We present a semibiological system employing electroactive bacteria integrated inside a porous and hydrophilic IO-ITO electrode architecture. The resulting biohybrid electrodes provide a platform to wire the bacteria’s intrinsic physiological functionalities with artificial electronics and allow a high degree of control over system configuration and operation. The biohybrid electrode attained a current density of 3 mA cm^−2^ at 0.1 V vs. SHE arising from microbial metabolism and represents a benchmark performance for microbial electrogenesis. Differential gene expression analysis revealed regulation of gene expression by *G. sulfurreducens* in response to changes in electron acceptors. The IO-ITO electrode also allowed *S. loihica* and *G. sulfurreducens* to metabolize in tandem and hence formed a syntrophic pathway for electrogenesis, which grants the system additional flexibility in using different electron donors to increase the stoichiometric electron production. Moreover, the resulting IO-ITO|*G. sulfurreducens* electrode can serve as a “living” cathode to reduce fumarate and GO with electrons supplied by an external electrochemical bias or by an irradiated photoanode. Coupling of microbial electrosynthesis with photoanodic water oxidation establishes the possibility of sustainable synthesis driven by sunlight. This biohybrid system synergizes metabolism with extracellular redox transformations via the electrical interplay at biointerfaces and can further be empowered with emerging methodologies in the realm of synthetic biology. With advancing genetic technologies, new biosynthetic pathways can be created and extended beyond the scope of naturally occurring metabolism. These will pave new avenues toward sustainable energy conversion and chemical synthesis.

### Data Availability.

Materials and methods, supplementary details, *SI Appendix*, Figs. S1–S14 and Table S1 and Movies S1–S3 are available in *SI Appendix*. Additional data (original data files and the dataset for the gene expression analysis) related to this publication are available at the University of Cambridge data repository (https://doi.org/10.17863/CAM.48465).

## Supplementary Material

Supplementary File

Supplementary File

Supplementary File

Supplementary File

Supplementary File
