# Supplementary material for: A three-dimensional hybrid electrode with electroactive microbes for efficient electrogenesis and chemical synthesis
Source: Proc Natl Acad Sci U S A. 2020 Feb 12;117(9):5074–80. doi: 10.1073/pnas.1913463117 (PMC7060665; doi:10.1073/pnas.1913463117)
Supplement: Supplementary File [file pnas.1913463117.sapp.pdf]

PNAS
www.pnas.org

Supplementary Information for

A three-dimensional hybrid electrode with electroactive microbes for efficient electrogenesis and chemical synthesis

Xin Fang, Shafeer Kalathil, Giorgio Divitini, Qian Wang, Erwin Reisner*

Erwin Reisner
Email: reisner@ch.cam.ac.uk

This PDF file includes:

- Materials and Methods
- Supplementary text
- Figures S1 to S14
- Tables S1
- Legends for Movies S1 to S3
- Legends for Dataset S1
- SI References

Other supplementary materials for this manuscript include the following:

- Movies S1 to S3
- Dataset S1

www.pnas.org/cgi/doi/10.1073/pnas.1913463117

Materials and Methods

Electrode preparation The IO-ITO electrodes were prepared by a co-assembly method.(S1, 2) Typically, ITO nanoparticles (Sigma-Aldrich, 20 mg) were dispersed in a mixture of methanol and water (11:1, *v*:*v*, 125 µL) and sonicated for 3 h. 10 µm polystyrene (PS) latex (0.75 mL, 2.5 wt% in water, Polysciences Inc.) was washed with water and methanol to remove the surfactant. The PS latex was centrifuged at 10,000 rpm for 3 min to remove the supernatant. The ITO nanoparticles dispersion was added to the PS beads and sonicated for 30 min in ice water (< 4 °C) to obtain a homogenous mixture. 15 µL of the PS-ITO mixture was dropcast onto ITO glass slides (Visiontek System Ltd., 1 cm × 2.5 cm × 0.11 cm, 12 Ω cm^{-2}) with a pre-defined area of 0.25 cm^2, and left drying in air for 30 min. The electrodes were then annealed at 500 °C in air for 20 min at a ramping rate of 1 °C min^{-1} from room temperature. The IO-ZrO_2 (10 µm) and IO-TiO_2 (10 µm) electrodes were prepared by a similar method with slight modifications: 5 mg TiO_2 nanoparticles (Evonik, Aeroxide® P25, 21 nm, anatalse/rutile (80:20)) were dispersed in 75 µL methanol/water mixture (6:1, *v*:*v*,), which was further mixed with PS beads centrifuged from 1 mL PS latex (2.5 wt% in water). 5 µL of the PS-TiO_2 mixture was dropcast onto ITO glass slides (0.25 cm^2).

The $BiVO_4$-CoO_x electrode was prepared according to a previously-reported method.(S3) Bismuth precursor thin films were first electrochemically deposited on FTO glass slides (Sigma Aldrich; 8 Ω sq^{-1}) in a three-electrode system consisting of a Ag/AgCl reference electrode (in 3 M NaCl solution) and a platinum counter electrode. $Bi(NO_3)_3$ solution (25 mL, 0.1 M, in acetic acid, pH 4.8) was mixed with *p*-benzoquinone (20 mL, 0.3 M, in ethanol) as the electrolyte. Electrodeposition was performed on an FTO glass slide with a pre-defined area of 1.0 cm^2 at 2.3 V vs. Ag/AgCl for 7 min at 25 °C using a potentiostat (MultiEmStat3+). The obtained bismuth precursor film was washed with water and dried in air, followed by dipcoating with vanadyl diacetylacetonate (0.2 M in dimethylsulfoxide/ethanol (1:1, *v*:*v*)). The electrode was then calcinated at 520 °C in air for 2 h at a ramping rate of 2 °C min^{-1}. Then the electrode was washed with NaOH (1 M) for 10 min and water to remove excess VO_x. The obtained $BiVO_4$ electrode was immersed in a $Co(NO_3)_2$ solution (20 mL, 10 mM, in 10 mM NH_3•H_2O, pH 8.4) for 30 min to load CoO_x. The $BiVO_4$-CoO_x electrode was then washed with water and annealed at 250 °C in air for 30 min at a ramping rate of 2 °C min^{-1}.

Culturing of bacteria *G. sulfurreducens* PCA (ATCC 51573) and *S. loihica* PV-4 were purchased from Leibniz-Institut DSMZ-Deutsche Sammlung von Mikroorganismen und Zellkulturen GmbH, Germany. *G. sulfurreducens* was cultured anaerobically in a medium

solution (DSMZ 826, see the following for details) using sodium acetate (20 mM) as the electron donor and sodium fumarate (50 mM) as the electron acceptor. The bacterial strain was inoculated in 20 mL of the medium solution in a sterilized vial and was purged with N_2:CO_2 (80:20 *v*:*v*%) for 1 h. The inoculated media were kept in a shaking incubator (30 °C, 180 rpm) for 5 days. *S. loihica* was cultured aerobically in Luria-Bertani medium solution (see the following for details) by keeping in a shaking incubator (30 °C, 180 rpm) overnight. The concentration of bacterial suspension was determined by measuring optical density (OD) at 600 nm using a UV–vis spectrometer (Varian Cary 50, Agilent Technologies). To suppress the outer-membrane *c*-type cytochromes without altering their genes, *G. sulfurreducens* was cultivated in the standard medium solution containing 2,2'-bipyridine (30 μM) as the iron chelator to limit the iron availability.(S4) *G. sulfurreducens* was first cultured in an iron-lacking medium at 30 °C for 5 days. Then 30 μM 2,2'-bipyridine was added into the medium and the bacteria were cultured anaerobically at 30 °C for another 5 days.

Components of the bacterium culturing mediums

(1) Medium for *Geobacter sulfurreducens* (DSMZ 826)

NH_4Cl	1.50 g
Na_2HPO_4	0.60 g
KCl	0.10 g
Trace element solution (see below)	10.00 mL
$NaHCO_3$	2.50 g
Vitamin solution (see below)	10.00 mL
Distilled water	980.00 mL

(2) Trace element solution

Nitrilotriacetic acid	1.50 g
$MgSO_4 \cdot 7H_2O$	3.00 g
$MnSO_4 \cdot H_2O$	0.50 g
NaCl	1.00 g
$FeSO_4 \cdot 7H_2O$	0.10 g
$CoSO_4 \cdot 7H_2O$	0.18 g
$CaCl_2 \cdot 2H_2O$	0.10 g
$ZnSO_4 \cdot 7H_2O$	0.18 g
$CuSO_4 \cdot 5H_2O$	0.01 g
$KAl(SO_4)_2 \cdot 12H_2O$	0.02 g
H_3BO_3	0.01 g

$Na_2MoO_4 \cdot 2H_2O$	0.01 g
$NiCl_2 \cdot 6H_2O$	0.03 g
$Na_2SeO_3 \cdot 5H_2O$	0.30 g
$Na_2WO_4 \cdot 2H_2O$	0.40 g
Distilled water	1000.00 mL

(3) Vitamin solution

Biotin	2.00 mg
Folic acid	2.00 mg
Pyridoxine-HCl	10.00 mg
Thiamine-HCl • $2H_2O$	5.00 mg
Riboflavin	5.00 mg
Nicotinic acid	5.00 mg
D-Ca-pantothenate	5.00 mg
Vitamin B12	0.10 mg
p-Aminobenzoic acid	5.00 mg
Lipoic acid	5.00 mg
H_3BO_3	0.01 g
Distilled water	1000.00 mL

(4) Luri-Bertani medium

Tryptone	10.00 g
Yeast extract	5.00 g
NaCl	10.00 g

Microbial electrogenesis The chronoamperometry and cyclic voltammetry were performed with a potentiostat (MultiEmStat3+) in a three-electrode system in a three-neck round bottom flask in a water bath (30 °C) under continuous stirring (200 rpm). A platinum mesh was used as the counter electrode and Ag/AgCl (in 3 M NaCl solution, +0.2 V vs. SHE) as the reference electrode. Sodium acetate (40 mM) was added into the medium solution (14 mL) as the electron donor. The electrolyte solution was purged with N_2:CO_2 (80:20 *v*:*v*%) for 40 min. *G. sulfurreducens* suspension (1 mL) was inoculated into the medium solution (final OD: 0.6 in total 15 mL) and purged for another 20 min. The IO-ITO electrodes were used as the working electrode and poised at different potentials (0.1–0.4 V vs. SHE). An ITO glass slide with a pre-defined area of 0.25 cm^2 was used for a comparison and an IO-ITO electrode without bacteria was used for control. Cyclic voltammetry was carried out after the anodic current reaching a plateau at a scan rate of

5 mV s^{-1}. To co-culture with *S. loihica*, sodium lactate (40 mM) was added in the medium solution (13 mL) as the electron donor to *S. loihica*, and purged with N_2:CO_2 (80:20 *v*:*v*%) for 40 min. *G. sulfurreducens* suspension (1 mL, final OD: 0.6) and *S. loihica* (1 mL, final OD: 0.6) were inoculated into the medium solution and purged for 20 min. The IO-ITO electrodes were used as the working electrode and poised at a potential of 0.4 V vs. SHE.

Protein quantification The proteins in the electrodes were quantified with a colorimetric assay following a previously-reported protocol.(S5) The bacteria-colonized electrode was immersed in a sodium dodecyl solution (5 mL, 10 wt%) solution at 99 °C for 15 min to extract proteins from the electrode. Then the solution was centrifuged (14,000 rpm, 10 min) to remove the impurities. The supernatant was used to quantify the protein using the Bio-Rad protein assay. Typically, 100 µL of the protein solution was added into a clean test tube, followed by adding 5 mL of the diluted dye reagent and incubating at 25 °C for 10 min. Then the light absorbance at 600 nm was measured using a UV–vis spectrometer. Bovine serum albumin was used as the standard protein to make the correlation curve. The protein concentration was calculated from the standard curve. Each protein solution was assayed in triplicate.

Differential gene expression analysis IO-ITO|*G. sulfurreducens* electrodes were prepared at different potentials following the method reported above. The plateau current density attained at 0.1 V and 0.4 V vs. SHE was 1.12 ± 0.05 mA cm^{-2} and 2.82 ± 0.25 mA cm^{-2}, respectively. The biohybrid electrode was scratched off the ITO glass slide and cells in electrodes were lysed with TissueLyser II immediately after the chronoamperometry was stopped. The mixture was sonicated for 1 min and centrifuged at 8000 ×g for 15 min. The resulting supernatant was used for RNA extraction. *G. sulfurreducens* anaerobically cultured in acetate (20 mM) and fumarate (50 mM) at 30 °C was used as control samples. RNA of *G. sulfurreducens* were extracted using an RNeasy protect bacteria mini kit (Qiagen, USA) at room temperature. The extracted RNA were eluted in nuclease free water. The RNA sequencing and data analysis was conducted by Cambridge Genomic Services (CGS). The quality control showed the q-score across all samples are above 30, signifying high sample quality. Reads were mapped with *G. sulfurreducens* reference genome using STAR v2.5.2a.(S6) Using the genes annotation defined in the *G. sulferreducens* gtf file from Ensembl Bacteria. The reads mapping into genomics features were counted using HTSeq v0.6.0. A feature is considered as the union of all gene's exons whose genomic coordinates are determined from the *G. sulfurreducens* gtf. Reads with a mapping quality less than 10, or those that map to multiple loci or to overlapping gene regions are discarded to avoid ambiguity and false

positives. Differential gene expression analysis was performed using the counted reads and the R package edgeR version 3.16.5 for the 3 pairwise comparisons.

Microbial electrosynthesis The electrosynthesis were performed with a potentiostat (MultiEmStat3+) in three-electrode configuration in a three-neck round bottom flask in a water bath (30 °C) under continuous stirring (200 rpm). A platinum mesh was used as the counter electrode and Ag/AgCl (in 3 M NaCl solution, +0.2 V vs. SHE) as the reference electrode. The IO-ITO|*G. sulfurreducens* electrode was replaced with a medium solution containing sodium fumarate (10 mM) or GO (0.1 mg mL^{-1}) and purged with N_2:CO_2 (80:20 *v*:*v*%) for 40 min. IO-ITO electrodes without bacteria were used for control experiments. The Faraday efficiency was determined by the ratio of electrons derived from succinate (two electrons per succinate) to the electrons consumed based on chronoamperometry. We note that such calculation might include charge contributions from reducing equivalents being stored in *G. sulfurreducens* during the anodic growth.(S7) A potential of −0.45 V vs. SHE was applied on the electrode for fumarate reduction and −0.3 V vs. SHE for GO reduction.

Microbial photoelectrosynthesis IO-TiO_2 (pore size: 10 μm; thickness: 40 μm; geometrical area: 0.25 cm^2) were cleaned with a UV/ozone cleaner for 15 min before use. The IO-TiO_2|**RuP** photoanode was prepared by immersing the IO-TiO_2 electrodes in [Ru^{II}bis(2,2'-bipyridine)(2,2'-bipyridine-4,4'-diylbis(phosphonic acid))]Br_2 (**RuP**,(S8) 0.25 mM in H_2O) for 16 h in the dark, followed by rinsing with water to remove weakly adsorbed dyes. Stepped chronoamperometry of the IO-TiO_2|**RuP** electrode was conducted in TEOA (25 mM, pH 7.2, in 0.1 M NaCl solution) in a N_2:CO_2 atmosphere (80:20, *v*:*v*%) with Pt and Ag/AgCl as the counter and reference electrode, respectively. The electrode was under periodic irradiation (10 s in light, 30 s in dark, I = 100 mW cm^{-2}, AM 1.5G) at stepped potentials from −0.7 V to 0.4 V vs. SHE every 40 s. The $BiVO_4$-CoO_x electrode (1.0 cm^2) was directly used as the photoanode without any pre-treatment. Linear sweep voltammetry of the $BiVO_4$-CoO_x electrode was carried out in a phosphate buffered saline solution (20 mM Na_2HPO_4, 3.6 mM KH_2PO_4, 5.4 mM KCl, 0.274 M NaCl, pH 7.3) in N_2:CO_2 (80:20 *v*:*v*%) under periodic irradiation (5 s in light, 5 s in dark, I = 100 mW cm^{-2}, AM 1.5G) at a scan rate of 5 mV s^{-1} with Pt and Ag/AgCl as the counter and reference electrode, respectively. The photoanode and the IO-ITO|*G. sulfurreducens* electrode were connected in a two-compartment, two-electrode photoelectrochemical cell separated by a Nafion membrane. A bare IO-ITO electrode without bacteria and an IO-ITO hybrid electrode with *G. sulfurreducens* killed by 0.1% glutaraldehyde were used for control experiments. TEOA (25 mM, pH 7.2, in 0.1 M NaCl solution) was used as the

electrolyte and electron donor for the IO-TiO_2|**RuP** electrode, whereas a phosphate buffered saline solution (20 mM Na_2HPO_4, 3.6 mM KH_2PO_4, 5.4 mM KCl, 0.274 M NaCl, pH 7.3) was used as the electrolyte for the $BiVO_4$-CoO_x electrode. Sodium fumarate (20 mM, in medium solution) was used as the substrate and electrolyte for the cathode. The photoelectrochemical cell was purged with N_2:CO_2 (80:20 *v*:*v*%) for 40 min. The photoelectrosynthesis was performed using simulated solar light (I = 100 mW cm^{-2}, AM 1.5G) (LOT Quantum Design) from a 150 W Xe lamp (Newport). Light intensity was calibrated by a thermal sensor (S302C (Thorlabs)) and power meter console (PM100D (Thorlabs)). Zero bias (U = 0) was applied between the photoanode and the cathode during the two-electrode photoelectrochemical experiment. Dark current was recorded for 30 min before and after irradiation for 24 h. Solution in the cathode chamber before and after the light experiment was extracted for product quantification.

Physical characterization SEM and STEM images were acquired on a scanning electrode microscope (TESCAN MIRA3) at an accelerating voltage of 5 kV and 30 kV respectively. X-ray microscopy image of the IO-ITO electrode was acquired on a 3D X-ray microscope (Zeiss Xradia 510 Versa). Cross-sectional SEM images were acquired on a focused ion beam-scanning electron microscope (FIB-SEM, ZEISS Crossbeam 540) at an acceleration voltage 1.6 kV. Serial sectioning was carried out using FIB milling at a 3 nA ion current and a slice thickness of 500 nm. Before SEM imaging, biohybrid electrodes were treated with 2.5 wt% glutaraldehyde and 2 wt% osmium tetraoxide, and then dehydrated with a series of ethanol solutions with increasing concentrations (30, 50, 70, 90 and 100%) and dried in air. The iron contents of bacteria and hybrid electrodes were determined by inductively coupled plasma-optical emission spectrometry (ICP-OES, Thermo scientific). Fluorescence images of IO-ITO|*G. sulfurreducens* electrodes were acquired on a confocal laser scanning microscope (Leica TCS SP8) using a 488 nm laser and a hybrid detector (600−650 nm). 100 µL of 5-cyano-2,3-ditolyl tetrazolium chloride (10 mM in medium solution) was dropcast on an IO-ITO|*G. sulfurreducens* electrode that was then incubated in the dark for 30 min at 25 °C. The AFM image was acquired on an atomic force microscope (NanoIR2, ANASYS Instrument) using a gold-coated silicon tip. ^{1}H NMR spectroscopy analysis was conducted on a Bruker 400 MHz NMR spectrometer in D_2O. Sodium 3-(trimethylsilyl)propionate-2,2,3,3-d^4 (TMSP-d^4, 1 mM) was used as the reference and internal standard for quantification. NMR spectra were processed with MestReNova v12.0. Raman spectra were recorded on a confocal Raman microscope (HORIBA LabRAM HR Evolution) with an excitation laser of 633 nm.

Statistical information All experiments were performed in three individual replicates unless otherwise mentioned. Data are presented in bar diagrams as mean ± standard error of the mean (s.e.m.). The mean values and standard errors of the mean were calculated from the number of repeats of independent experiments. Statistical analyses were conducted using GraphPad Prism v.6.0 g. Statistical significance was determined by one-sided analysis of variance (ANOVA) for multiple groups of samples and Student's t tests for unpaired two samples. $P < 0.05$ was deemed statistically significant. Significance values: *n.s.*, not significant ($P > 0.05$), ****$P < 0.0001$.

Supplementary text

(1) Significance summary of differential gene expression analysis

Comparison	Total genes	FDR < 0.05 genes		
		Total	Up-regulation	Down-regulation
0.1 V vs. 0.4 V	3586	19	1	18
0.1 V vs. Ctrl	3612	978	250	728
0.4 V vs. Ctrl	3602	907	234	673

(2) Three genes encoding proteins involved in extracellular electron transfer

Genes	Proteins	0.1 V vs. Ctrl		0.4 V vs. Ctrl		0.1 V vs. 0.4 V	
		Log_2FC	FDR	Log_2FC	FDR	Log_2FC	FDR
GSU2737	OmcB	0.83	0.003	1.19	<0.0001	-	n.s.*
GSU0466	MacA	0.383	0.006	0.29	0.034	-	n.s.
GSU3304	OmpJ	0.434	0.020	0.43	0.018	-	n.s.

*Not significant

(3) Extended discussion of differential gene expression analysis

Microorganisms survive and thrive under different environments thanks to their acute and flexible regulatory mechanisms, which enable them to maintain their homeostasis in response to environmental variations. Through various sensing and transduction pathways, extracellular changes can be translated into intracellular signals that induce transcriptional responses.(S9) RNA sequencing is widely employed for gene expression profiling,(S10) which can provide a global view on microbial strategies to cope with environmental perturbations.

G. sulfurreducens can use various forms of soluble and insoluble electron acceptors.(S11) Soluble electron acceptors such as fumarate can diffuse into a cell and participate in intracellular metabolism, whereas insoluble electron acceptors such as metal oxides cannot permeate through the cellular membrane and can only be utilized for anaerobic respiration by extracellular electron transfer. In a biohybrid system, an electrode with a poised electrochemical potential substitute for natural minerals as an inexhaustible electron sink, which forms the basis of microbial electrogenesis. When *G. sulfurreducens* is transferred to a new environment deprived of soluble electron acceptors (fumarate), bacteria will regulate gene expression to align their metabolism with the physiological needs of using an electrode as the electron acceptor. Although their cellular regulation has not been completely deciphered due to many genes with unknown functionality,(S12) several genes that are found responsible for extracellular electron transport could shed light on part of their adaptation strategies. Genome-wide study has

identified 111 gene encoding putative Cyt *c* (109 of them found in this study) in *G. sulfurreducens*,(S6) but only 16 multi-haem Cyt *c* were found essential for extracellular electron transfer.(S13)

Here, we conducted differential gene expression analysis for *G. sulfurreducens* grown with fumarate (the control) and cultured on electrodes with different potentials (0.1 V and 0.4 V vs. SHE). Amongst more than 3500 genes sequenced, ~25% of genes were induced with significant changes (FDR < 0.05) in expression on electrodes, with ~75% of them down-regulated (see Significance summary). The down regulation of genes indicates that bacteria saved more energy when respiring on electrodes.(S14) However, there were much less differences in gene expression between electrodes at different potentials, suggesting potential variations cannot stimulate extensive transcriptional responses.

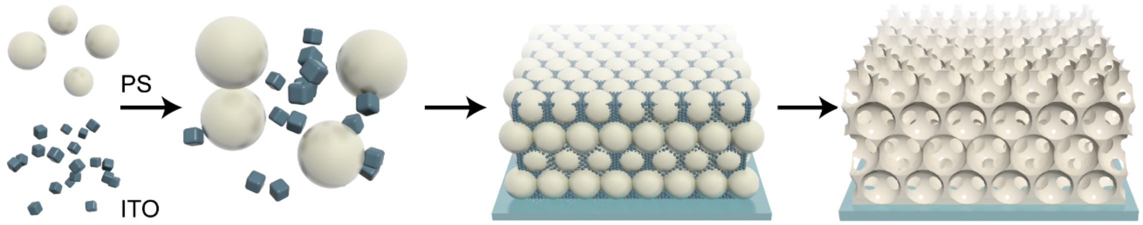

Fig. S1. Co-assembly method to prepare the IO-ITO electrodes. PS beads (10 μm) were mixed with ITO nanoparticles (average size < 50 nm) and an aliquot of the colloidal mixture was dropcast on an ITO glass substrate. The IO-ITO electrodes were obtained by annealing at 500 °C to remove the PS template and sinter the mesoporous ITO skeleton (see Methods for details). The same procedure has also been employed for IO-TiO_2 and IO-ZrO_2 electrodes used in this study.

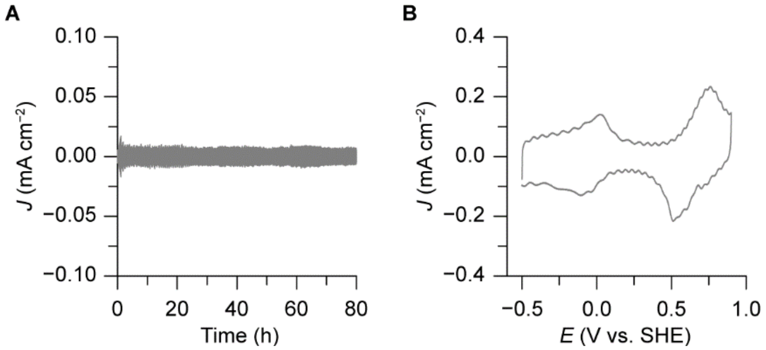

Fig. S2. Chronoamperometry (**A**) and CV scan (**B**) of the IO-ITO electrode with heat-killed *G. sulfurreducens*. *G. sulfurreducens* (OD 0.6) was inactivated by heating at 150 °C for 1 h in an autoclave. Conditions: *E* = 0.4 V (vs. SHE), 40 mM acetate, pH 7.4; CV: scan rate: 5 mV s^{-1}, 30 °C, purged with N_2:CO_2 (80:20, *v*:*v*%).

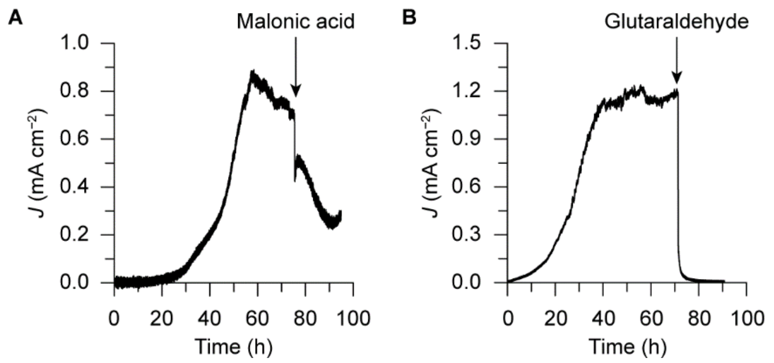

Fig. S3. A. Effect of a metabolism inhibitor on *G. sulfurreducens*. The black arrow indicates the addition of malonic acid (10 mM) into the electrolyte solution. Malonic acid inhibits the TCA cycle and partially suppresses the metabolism of *G. sulfurreducens*. **B.** Effect of a biocide to *G. sulfurreducens*. The black arrow indicates the addition of glutaraldehyde (0.1%), a typical biocide, into the electrolyte solution. Conditions: E = 0.4 V vs. SHE, 40 mM acetate, pH 7.4, 30 °C, purged with N_2:CO_2 (80:20, *v*:*v*%).

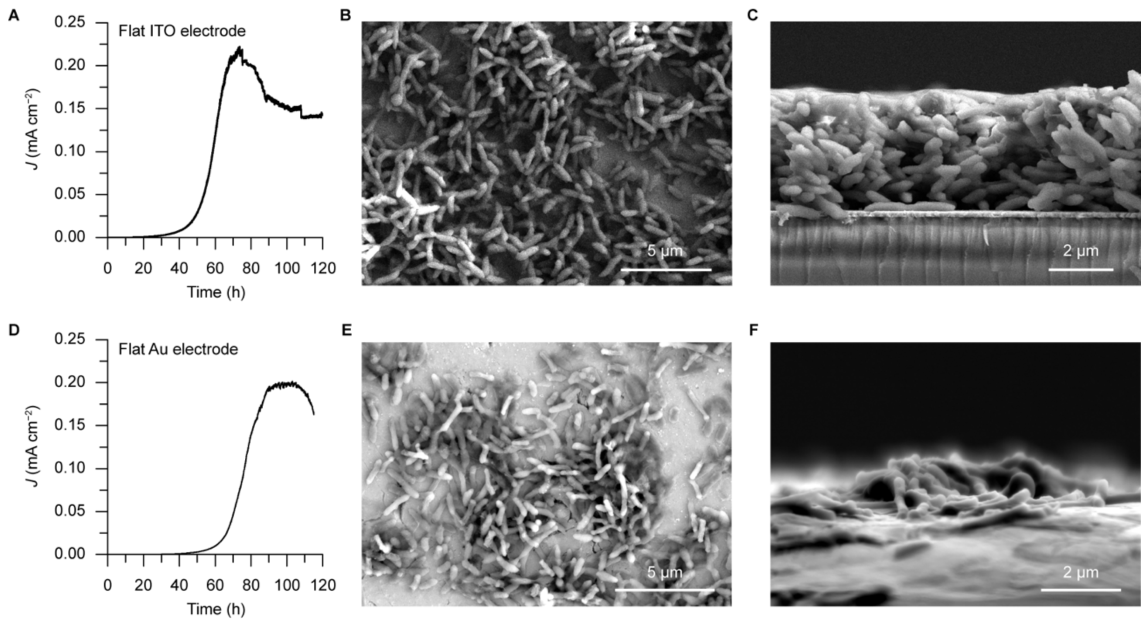

Fig. S4. A. Chronoamperometry of a flat ITO-coated glass electrode with *G. sulfurreducens*. **B,C.** SEM images of the *G. sulfurreducens* on a flat ITO glass electrode from a top (**A**) and a side (**B**) view. **D.** Chronoamperometry of a flat Au electrode with *G. sulfurreducens*. **E,F.** SEM images of the *G. sulfurreducens* on a flat Au electrode from a top (**E**) and a side (**F**) view. Conditions: E = 0.1 V vs. SHE, 40 mM acetate, pH 7.4; 30 °C, purged with N_2:CO_2 (80:20, *v*:*v*%).

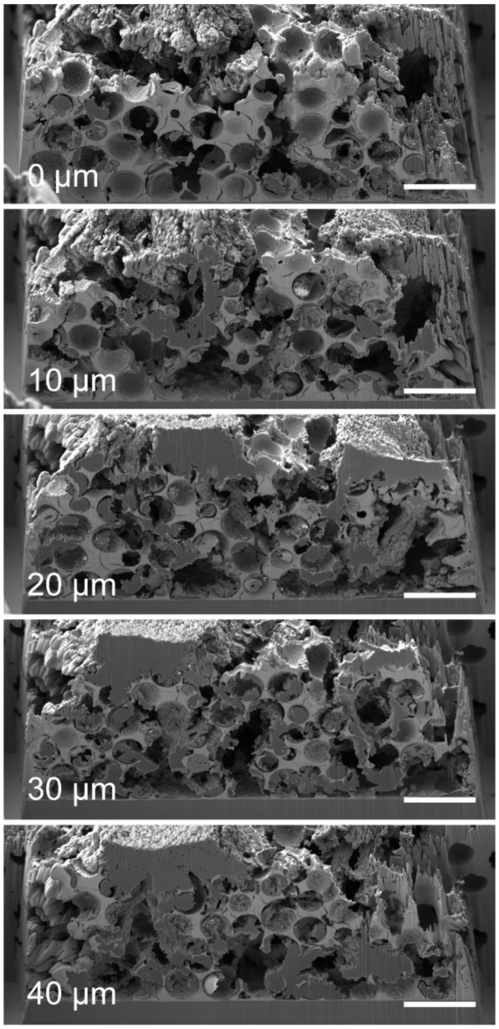

Fig. S5. Serial cross-sectional SEM images of an IO-ITO|*G. sulfurreducens* hybrid electrode prepared at 0.1 V vs. SHE for 80 h. Cross-sectional views of every 10 μm are displayed (see **Movie S3**). Scale bars: 20 μm.

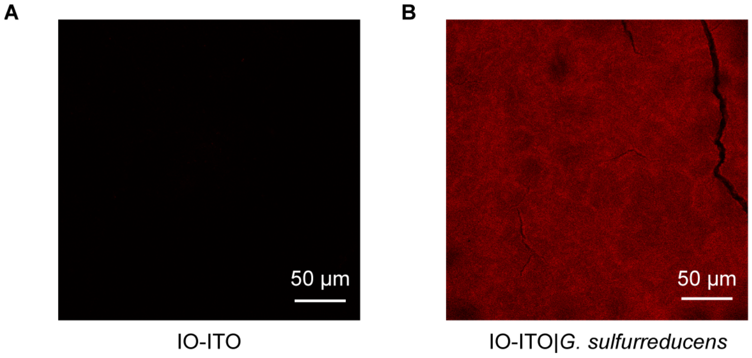

Fig. S6. CLSM images of a bare IO-ITO electrode (**A**) and IO-ITO|*G. sulfurreducens* hybrid electrode (**B**). The electrodes were stained with 5-cyano-2,3-ditolyl tetrazolium chloride (CTC, 10 mM) and incubated in the dark for 30 min at 25 °C. As conventional fluorescent dyes (such as SYTO 9 and propidium iodide) adsorbed on the IO-ITO scaffold would be unable to differentiate between live and dead cells, we used an alternative dye to assess the bacterial viability. CTC is a soluble non-fluorescent compound whereas its reduced form, CTC formazan, is insoluble and produces red fluorescence. Living bacteria respiring via the electron transport chain adsorb CTC dyes and reduce them into insoluble CTC formazan that precipitates in the cell. Dead bacteria and IO-ITO scaffolds cannot reduce CTC, thereby producing much less fluorescence.(S15) This method allows us to assess the bacterial viability inside the IO-ITO electrode by means of evaluating their respiratory activity, without being interfered by the scaffold.

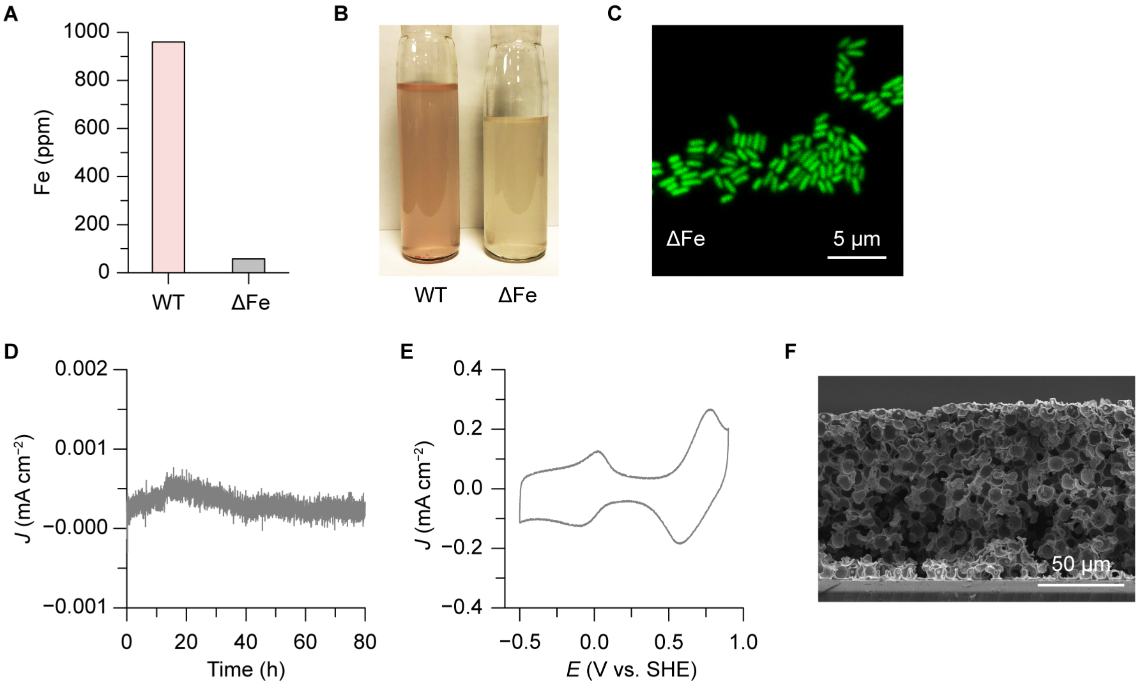

Fig. S7. Control experiments with Fe-depleted *G. sulfurreducens*. **A.** The Fe quantity the wild-type (WT) and Fe-depleted (ΔFe) *G. sulfurreducens,* determined by ICP-OES. **B.** Photographs of the WT and ΔFe *G. sulfurreducens.* The reddish color disappeared for ΔFe *G. sulfurreducens* due to lack of iron. **C.** CLSM image of the ΔFe *G. sulfurreducens* that was stained with the LIVE/DEAD BacLight bacterial viability kit (L7007) to distinguish the live (green) and dead (red) cells. The CLSM image shows a majority of ΔFe bacteria were live. Excitation: 476 nm; Emission: 480–520 nm (SYTO 9) and 610–660 nm (propidium iodide). **D**,**E.** Chronoamperometry and CV scan of the IO-ITO electrode with ΔFe *G. sulfurreducens*. **F.** SEM image of the IO-ITO electrode with ΔFe *G. sulfurreducens.* Bacteria without Cyt *c* were not able to respire with the IO-ITO scaffold, thereby not forming biofilm within the electrode. Conditions: E = 0.4 V vs. SHE, 40 mM acetate, pH 7.4; CV: scan rate: 5 mV s^{-1}; 30 °C, purged with N_2:CO_2 (80:20, *v*:*v*%).

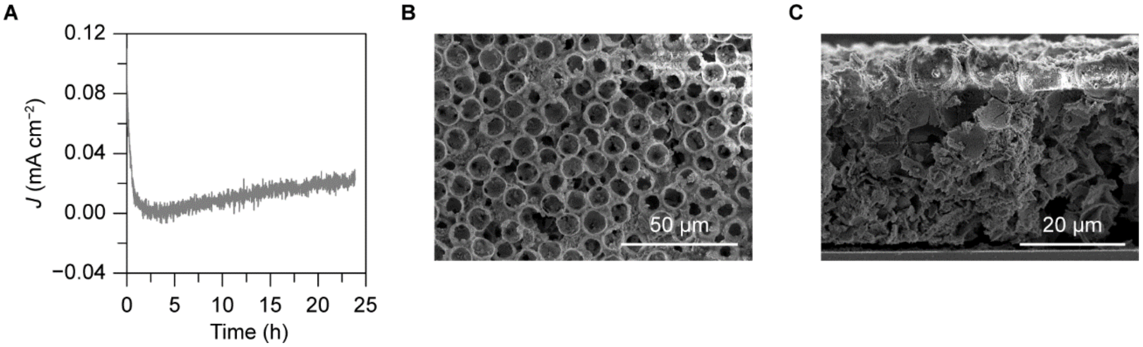

Fig. S8. Control experiment with IO-ZrO_2 electrode (Geometrical area: 0.25 cm^2; pore size: 10 μm; thickness: 50 μm). **A.** Chronoamperometry of the IO-ZrO_2 electrode with *G. sulfurreducens*. **B,C.** SEM images of the IO-ZrO_2 electrode from the top (**B**) and side (**C**) view of the IO-ZrO_2 electrode after anodic culturing. There were no bacteria colonizing within the non-conductive scaffold. Conditions: E = 0.4 V vs. SHE, 40 mM acetate, pH 7.4; 30 °C, purged with N_2:CO_2 (80:20, *v*:*v*%). The non-conductive IO-ZrO_2 scaffold was prepared on an ITO glass substrate following a similar co-assembly method using 10 μm PS beads and ZrO_2 nanoparticles (20–30 nm).

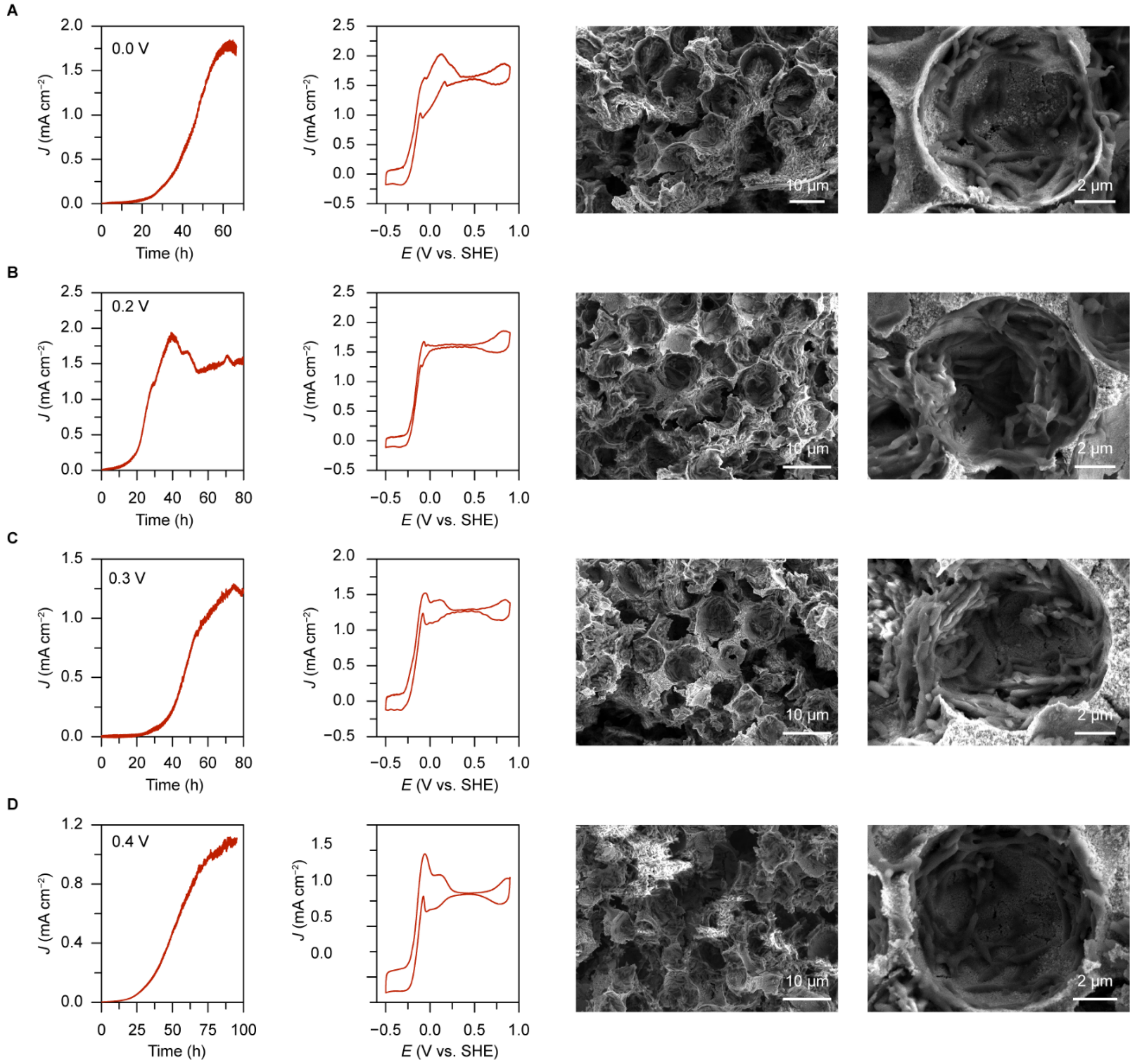

Fig. S9. Current density, CV scans and SEM images of IO-ITO|*G. sulfurreducens* electrodes at different potentials with acetate (40 mM, pH 7.4). **A.** 0.0 V vs. SHE; **B.** 0.2 V vs. SHE; **C.** 0.3 V vs. SHE; **D.** 0.4 V vs. SHE. Scan rate: 5 mV s^{-1}. All the electrochemical experiments were performed prepared 30 °C in N_2:CO_2 (80:20, *v*:*v*%).

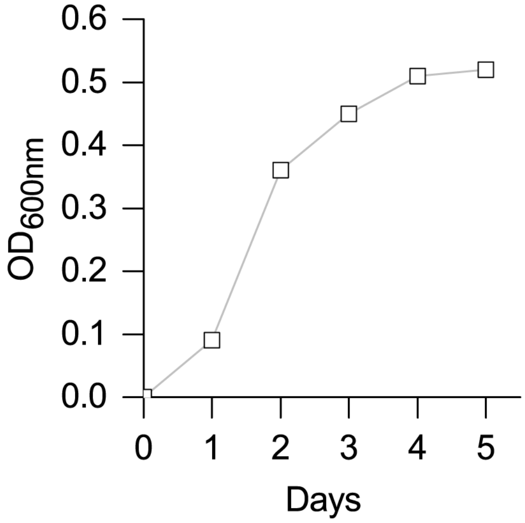

Fig. S10. The optical density at 600 nm ($OD_{600\ nm}$) of planktonic *G. sulfurreducens* cultured with acetate (20 mM) and fumarate (50 mM) in N_2:CO_2 (80:20 *v*:*v*%) at 30 °C.

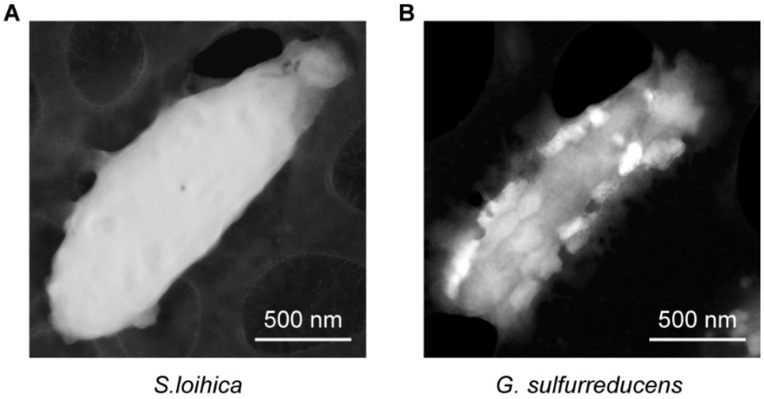

Fig. S11. STEM images of a *S. loihica* (**A**) and *G. sulfurreducens* (**B**).

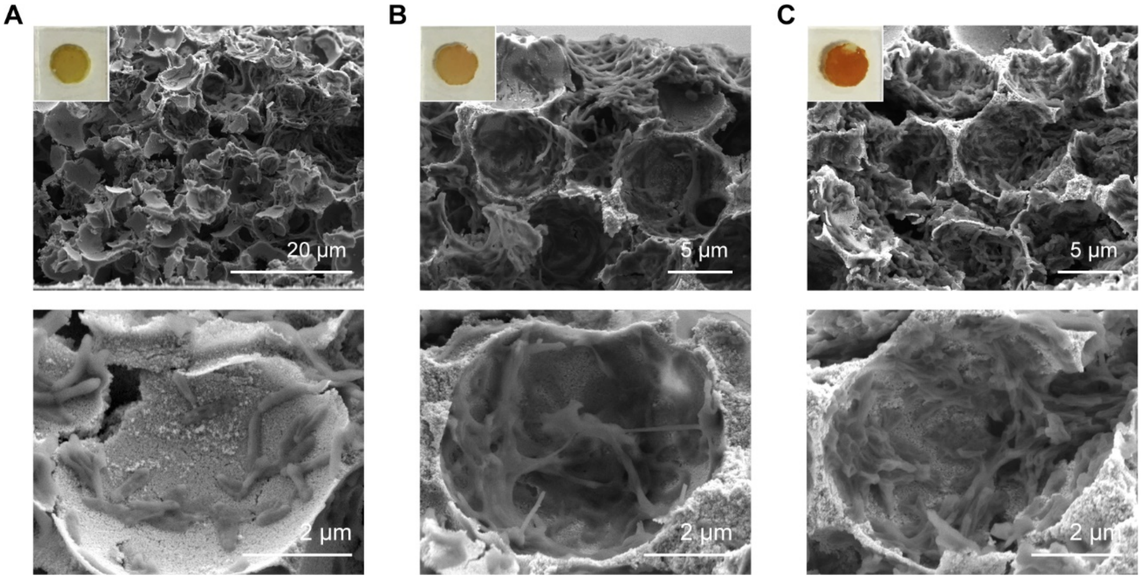

Fig. S12. Cross-sectional SEM images of IO-ITO|*G. sulfurreducens* (**A**), IO-ITO|*S. loihica* (**B**), and IO-ITO|mixed-cultures (**C**) The inserted images show the photographs of the hybrid electrodes. The SEM images along with photographs representatively reflect the bacterial colony in different electrodes, but they are unable to distinguish *G. sulfurreducens* and *S. loihica* due to similar morphologies. All these hybrid electrodes were prepared at 0.4 V vs. SHE with lactate (40 mM, pH 7.4) at 30 °C in N_2:CO_2 (80:20, *v*:*v*%).

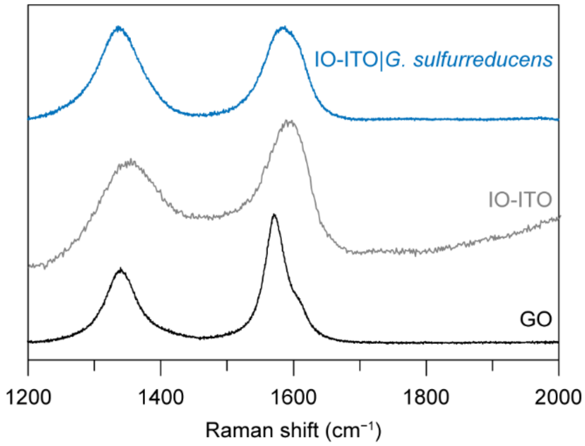

Fig. S13. Raman spectra of the GO before and after reduced by an IO-ITO electrode and an IO-ITO|*G. sulfurreducens* hybrid electrode at −0.3 V vs. SHE. The intensity ratio of D (~1350 cm^{-1}) and G band (~1580 cm^{-1}): 0.57 (GO), 0.87 (IO-ITO), 1.00 (IO-ITO|*G. sulfurreducens*).

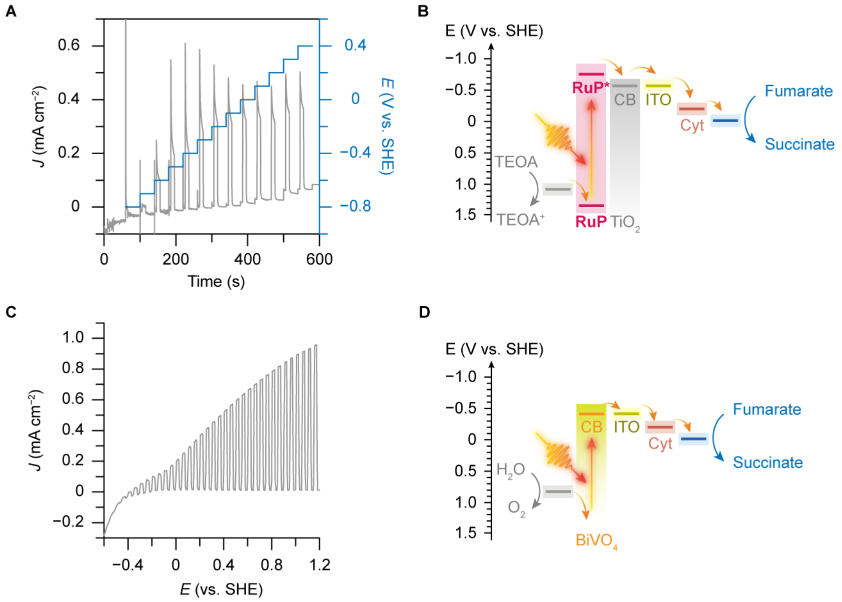

Fig. S14. A. Stepped chronoamperometry of the IO-TiO_2|**RuP** photoanode in TEOA (25 mM, pH 7.2), with Pt and Ag/AgCl as the counter and reference electrode, respectively. The electrode was under periodic irradiation (10 s in light, 30 s in dark, I = 100 mW cm^{-2}, AM 1.5G) at stepped potentials from −0.7 V to 0.4 V vs. SHE. The experiment was performed in N_2:CO_2 (80:20, *v*:*v*%). **B.** Energy level diagram of an IO-TiO_2|**RuP** photoanode coupled with an IO-ITO|*G. sulfurreducens* cathode with respect to an electrochemical potential scale at pH 7.0. Energy levels of TiO_2 and **RuP** are taken from Ref. (S16). As the mechanism of electron intake by bacterium remains elusive, here we tentatively assume electrons are transferred inward via *c*-type cytochromes with redox potentials centered at −0.2 V vs. SHE.(S17, 18) The reduction potential of fumarate (0.03 V vs. SHE) is sourced from Ref. (S19). **C.** Linear sweep voltammetry trace of the $BiVO_4$-CoO_x electrode in phosphate buffered saline (20 mM Na_2HPO_4, pH 7.3) under periodic irradiation (5 s in light, 5 s in dark, I = 100 mW cm^{-2}, AM 1.5G). Scan rate: 5 mV s^{-1}. Pt and Ag/AgCl were employed as the counter and reference electrode, respectively. The experiment was performed in N_2:CO_2 (80:20, *v*:*v*%). **D.** Energy level diagram of a $BiVO_4$-CoO_x photoanode coupled with an IO-ITO|*G. sulfurreducens* cathode with respect to an electrochemical potential scale at pH 7.0. The conduction band (CB) edge of $BiVO_4$ was taken from Ref. (S20)

Table S1. Summary of the electrogenic performance of various microbial electrochemical systems

Microorganisms	Electrodes	Thickness (mm)	*E* (V vs. SHE)	*J*(Areal) (mA cm^{-2})	*J*(Volumetric) (mA cm^{-3})	Ref.
G. sulfurreducens	3D-ITO	0.06	0.1	3.0	500	This work
G. sulfurreducens	3D-ITO	0.06	0.4	1.0	166	This work
G. sulfurreducens	3D-CNT	1.3	0.2	2.45	19	(S21)
G. sulfurreducens	Carbon fiber fabric	1.5	0.4	3.0	20	(S22)
G. sulfurreducens	3D Ti_4O_7 scaffold	13.5	0.4	12.87	9.5	(S23)
G. sulfurreducens	RGO-Vitamin B_2	-	0.3	0.25	-	(S24)
G. sulfurreducens	TiO_2 nanoparticles	-	0.24	0.33	-	(S25)
G. sulfurreducens	3D Ti_4O_7 scaffold	11	0.4	21.3	20	(S26)
S. loihica	ITO nanowires (10 μm)	0.01	0.4	0.025	2.5	(S27)
S. loihica	ITO glass with EDTA	-	0.4	0.024	-	(S28)
S. oneidensis	3D graphene aerogel	15	MFC	0.49	0.3	(S29)
Mixed cultures	Gold plate	0.13	0.4	1.18	9.4	(S30)
	Silver plate	0.15	0.4	1.12	7.5	
	Graphite	0.12	0.4	1.10	9.2	
	Copper plate	0.25	0	1.52	6.1	
	Nickel	0.08	0	0.38	5.1	
Mixed cultures	Layered corrugated carbon	18	0.4	39.1	21.7	(S31)
Mixed cultures	3D carbon felt		0.4	0.35		(S32)
Mixed cultures	Activated carbon-CaS		0.45	2.13		(S33)
	Activated carbon-FeS		0.45	1.07		
Mixed cultures	RGO-FeS_2	-	0.4	0.805	-	(S34)
Mixed cultures	Carbon foam	2.1	0.4	4	18.7	(S35)
Mixed cultures	Graphene sponge	4	0.4	1.07	2.7	(S36)

Movie S1.

X-ray microscopy image of the IO-ITO electrode

Movie S2.

Serial cross-sectional SEM images of an IO-ITO electrode acquired from FIB-SEM.

Movie S3.

Serial cross-sectional SEM images of an IO-ITO|*G. sulfurreducens* hybrid electrode prepared at 0.1 V vs. SHE for 80 h.

Dataset S1.

Results of differential gene expression analysis

Supplementary References

S1. Fang X*, et al.* Structure–activity relationships of hierarchical three-dimensional electrodes with photosystem II for semiartificial photosynthesis. *Nano Lett.* **19**, 1844-1850 (2019).

S2. Zhang JZ*, et al.* Photoelectrochemistry of photosystem II in vitro vs in vivo. *J. Am. Chem. Soc.* **140**, 6-9 (2018).

S3. Zhong M*, et al.* Bulky crystalline BiVO4 thin films for efficient solar water splitting. *J. Mater. Chem. A* **4**, 9858-9864 (2016).

S4. Estevez-Canales M*, et al.* A severe reduction in the cytochrome C content of Geobacter sulfurreducens eliminates its capacity for extracellular electron transfer. *Environ Microbiol Rep.* **7**, 219-226 (2015).

S5. Bradford MM A rapid and sensitive method for the quantitation of microgram quantities of protein utilizing the principle of protein-dye binding. *Anal. Biochem.* **72**, 248-254 (1976).

S6. Methé BA*, et al.* Genome of Geobacter sulfurreducens: Metal Reduction in Subsurface Environments. *Science* **302**, 1967-1969 (2003).

S7. Freguia S, Rabaey K, Yuan Z, & Keller J Electron and carbon balances in microbial fuel cells reveal temporary bacterial storage behavior during electricity generation. *Environ. Sci. Technol.* **41**, 2915-2921 (2007).

S8. Trammell SA*, et al.* Sensitization of TiO2 by Phosphonate-Derivatized Proline Assemblies. *Inorg. Chem.* **38**, 3665-3669 (1999).

S9. López-Maury L, Marguerat S, & Bähler J Tuning gene expression to changing environments: from rapid responses to evolutionary adaptation. *Nat. Rev. Genet.* **9**, 583 (2008).

S10. Wang Z, Gerstein M, & Snyder M RNA-Seq: a revolutionary tool for transcriptomics. *Nat. Rev. Genet.* **10**, 57-63 (2009).

S11. Lovley DR*, et al.* (2011) Geobacter: The Microbe Electric's Physiology, Ecology, and Practical Applications. *Advances in Microbial Physiology*, ed Poole RK (Academic Press), Vol 59, pp 1-100.

S12. Chan CH, Levar CE, Jiménez-Otero F, & Bond DR Genome Scale Mutational Analysis of Geobacter sulfurreducens Reveals Distinct Molecular Mechanisms

for Respiration and Sensing of Poised Electrodes versus Fe(III) Oxides. *J. Bacteriol.* **199**, e00340-00317 (2017).

S13. Shi L, Squier TC, Zachara JM, & Fredrickson JK Respiration of metal (hydr)oxides by Shewanella and Geobacter: a key role for multihaem c-type cytochromes. *Mol. Microbiol.* **65**, 12-20 (2007).

S14. Wagner A Energy Constraints on the Evolution of Gene Expression. *Mol. Biol. Evol.* **22**, 1365-1374 (2005).

S15. Kobayashi T*, et al.* Use of 5-cyano-2,3-ditolyl-tetrazolium chloride staining as an indicator of biocidal activity in a rapid assay for anti-Acanthamoeba agents. *J. Clin. Microbiol.* **50**, 1606-1612 (2012).

S16. Warnan J*, et al.* Solar H2 evolution in water with modified diketopyrrolopyrrole dyes immobilised on molecular Co and Ni catalyst–TiO2 hybrids. *Chem. Sci.* **8**, 3070-3079 (2017).

S17. Rosenbaum M, Aulenta F, Villano M, & Angenent LT Cathodes as electron donors for microbial metabolism: Which extracellular electron transfer mechanisms are involved? *Bioresour. Technol.* **102**, 324-333 (2011).

S18. Zacharoff LA & El-Naggar MY Redox conduction in biofilms: From respiration to living electronics. *Curr. Opin. Electrochem.* **4**, 182-189 (2017).

S19. Thauer RK, Jungermann K, & Decker K Energy conservation in chemotrophic anaerobic bacteria. *Bacteriol. Rev.* **41**, 100-180 (1977).

S20. Park Y, McDonald KJ, & Choi K-S Progress in bismuth vanadate photoanodes for use in solar water oxidation. *Chem. Soc. Rev.* **42**, 2321-2337 (2013).

S21. Katuri K*, et al.* Three-dimensional microchanelled electrodes in flow-through configuration for bioanode formation and current generation. *Energy Environ. Sci.* **4**, 4201-4210 (2011).

S22. Chen S*, et al.* Electrospun and solution blown three-dimensional carbon fiber nonwovens for application as electrodes in microbial fuel cells. *Energy Environ. Sci.* **4**, 1417-1421 (2011).

S23. Massazza D, Parra R, Busalmen JP, & Romeo HE New ceramic electrodes allow reaching the target current density in bioelectrochemical systems. *Energy Environ. Sci.* **8**, 2707-2712 (2015).

S24. Yu S-S*, et al.* Framework of cytochrome/vitamin B2 linker/graphene for robust microbial electricity generation. *ACS Appl. Mater. & Interfaces* **10**, 35090-35098 (2018).

S25. Zhou S, Tang J, Yuan Y, Yang G, & Xing B TiO2 nanoparticle-induced nanowire formation facilitates extracellular electron transfer. *Environ. Sci. Technol. Lett.* **5**, 564-570 (2018).

S26. Massazza D, Busalmen JP, Parra R, & Romeo HE Layer-to-layer distance determines the performance of 3D bio-electrochemical lamellar anodes in microbial energy transduction processes. *J. Mater. Chem. A* **6**, 10019-10027 (2018).

S27. Bian R*, et al.* Highly boosted microbial extracellular electron transfer by semiconductor nanowire array with suitable energy level. *Adv. Funct. Mater.* **28**, 1707408 (2018).

S28. Wang Y*, et al.* Facile one-step strategy for highly boosted microbial extracellular electron transfer of the genus Shewanella. *ACS Nano* **10**, 6331-6337 (2016).

S29. Zhao S*, et al.* Three-dimensional graphene/Pt nanoparticle composites as freestanding anode for enhancing performance of microbial fuel cells. *Sci. Adv.* **1**, e1500372 (2015).

S30. Baudler A, Schmidt I, Langner M, Greiner A, & Schröder U Does it have to be carbon? Metal anodes in microbial fuel cells and related bioelectrochemical systems. *Energy Environ. Sci.* **8**, 2048-2055 (2015).

S31. Chen S*, et al.* Layered corrugated electrode macrostructures boost microbial bioelectrocatalysis. *Energy Environ. Sci.* **5**, 9769-9772 (2012).

S32. Blanchet E, Erable B, De Solan M-L, & Bergel A Two-dimensional carbon cloth and three-dimensional carbon felt perform similarly to form bioanode fed with food waste. *Electrochem. Commun.* **66**, 38-41 (2016).

S33. Yasri NG & Nakhla G Impact of interfacial charge transfer on the start-up of bioelectrochemical systems. *J. Environ. Chem. Eng.* **5**, 3640-3648 (2017).

S34. Wang R*, et al.* FeS2 nanoparticles decorated graphene as microbial fuel cell anode achieving high power density. *Adv. Mater.* **30**, 1800618 (2018).

S35. Chen S*, et al.* Reticulated carbon foam derived from a sponge-like natural product as a high-performance anode in microbial fuel cells. *J. Mater. Chem.* **22**, 18609-18613 (2012).

S36. Xie X*, et al.* Graphene–sponges as high-performance low-cost anodes for microbial fuel cells. *Energy Environ. Sci.* **5**, 6862-6866 (2012).

End of Supplementary Information
